# The Governance of Core Competencies for Public Health: A Rapid Review of the Literature

**DOI:** 10.3389/phrs.2023.1606110

**Published:** 2023-09-12

**Authors:** Harman Singh Sandhu, Victoria Otterman, Lynda Tjaden, Rosemarie Shephard, Emma Apatu, Erica Di Ruggiero, Richard Musto, Jasmine Pawa, Malcolm Steinberg, Claire Betker

**Affiliations:** ^1^ National Collaborating Centre for Determinants of Health, St. Francis Xavier University, Antigonish, NS, Canada; ^2^ Dalla Lana School of Public Health, University of Toronto, Toronto, ON, Canada; ^3^ Public Health Agency of Canada (PHAC), Ottawa, ON, Canada; ^4^ Department of Health Research Methods, Evidence & Impact, Faculty of Health Sciences, McMaster University, Hamilton, ON, Canada; ^5^ Canadian Public Health Association, Ottawa, ON, Canada; ^6^ Division of Clinical Sciences, Northern Ontario School of Medicine (NOSM) University, Sudbury, ON, Canada; ^7^ Faculty of Health Sciences, Simon Fraser University, Burnaby, BC, Canada

**Keywords:** competencies, public health, education, workforce, governance

## Abstract

Core competencies for public health (CCPH) define the knowledge, skills, and attitudes required of a public health workforce. Although numerous sets of CCPH have been established, few studies have systematically examined the governance of competency development, review, and monitoring, which is critical to their implementation and impact. This rapid review included 42 articles. The findings identified examples of collaboration and community engagement in governing activities (e.g., using the Delphi method to develop CCPH) and different ways of approaching CCPH review and revision (e.g., every 3 years). Insights on monitoring and resource management were scarce. Preliminary lessons emerging from the findings point towards the need for systems, structures, and processes that support ongoing reviews, revisions, and monitoring of CCPH.

## Introduction

According to the Chief Public Health Officer of Canada’s 2021 report, public health systems aim to enhance population health, promote health equity, and protect against health emergencies [1]. The public health workforce, which is diverse and interprofessional, is a critical building block of the public health system [1]. The workforce can be supported by sets of competencies that represent a combination of knowledge, skills, and attitudes deemed necessary for public health practice [2,3]. Well-defined competencies have the potential to improve public health system performance via a strong, capable, and guided workforce [4].

There has been a growing interest in identifying and revising core competencies for public health (CCPH) over the past few decades [5–7]. CCPH account for the expansive scope of public health and transcend roles, disciplines, and settings by providing the foundation for effective public health practice and the use of a public health approach [2]. Sets of CCPH have been developed and published in many jurisdictions (e.g., Europe, Canada, Australia, the United Kingdom [UK], the United States of America [USA], and New Zealand) [2, 8–12]. In 2022, the World Health Organization (WHO) launched a project to identify the competencies required by the *worldwide* public health workforce to deliver essential public health functions in a post-COVID-19 era [13]. Defining CCPH is an ongoing effort that requires systems and structures to support the review of existing competencies and the development of new ones as population health needs and approaches evolve [5]. We believe that governance is a key factor in supporting ongoing CCPH-related activities.

Governance, in the context of public health systems, refers to how “different public, non-governmental, or private actors work together to support communities in preventing disease and achieving health, wellbeing, and health equity” (14 p1). Policy development, resource stewardship, partner and community engagement, continuous improvement, and oversight are considered to be the functions of public health governance [14, 15] and are guided by principles such as direction and priority-setting, transparency, accountability, inclusion, equity, collaboration, and sustainability [16–26]. These functions and principles of governance can be used to examine how structures and processes support the development, implementation, and sustainability of CCPH-related activities.

The COVID-19 pandemic has sparked a renewed focus on strengthening public health systems [1, 27] and there have been numerous calls to modernize the *Core Competencies for Public Health in Canada*, originally published by the Public Health Agency of Canada (PHAC) in 2008 [1, 2, 28–31]. The absence of governance structures and processes to support implementation as well as regular review and revision of the PHAC-CCPH has been identified as a particular gap. Our work aims to support modernization efforts by first conducting a jurisdictional scan to learn how sets of CCPH are governed worldwide. We drew from the literature on governance [14–26, 32–38] to design the following research question: How have CCPH been developed, reviewed, and monitored? The findings will add to the evidence as no previous studies have systematically examined this aspect of CCPH and inform considerations for a governance approach for the PHAC-CCPH.

## Methods

### Study Design

Our protocol was informed by rapid review guidelines from the National Collaborating Centre (NCC) for Methods and Tools [39] and WHO [40]. A rapid review is a relatively quick assessment of “what is already known about a policy or practice issue” [41, p95] and uses systematic methods while promoting flexibility and timeliness [39, 40]. This study was conducted as part of a broader collaboration between PHAC and the NCCs for Public Health. An NCC project team (HS, LT, CB) led the study with input from PHAC (VO, RS, LF, JU, EP) and an advisory committee of Canadian public health academics and practitioners (EA, EDR, RM, JP, MS). Regular meetings were held to discuss the study design, methodology, and emerging findings, helping to strengthen the relevance and potential applicability of this study. Procedural research ethics board approval was not required as this study was based on publicly available literature [42].

### Search Strategy

We collaborated with an information specialist (KC) to design and conduct the search of academic and grey literature. Four concepts were identified from which expansive search terms were developed: 1) competencies; 2) public health; 3) core; and 4) governance. Three databases were searched. The National Library of Medicine’s *PubMed* and USA Department of Education’s *ERIC* databases were searched on July 27, 2022, and EBSCO’s *CINAHL Plus* was searched on August 1, 2022. The retrieval of articles was limited to those published in English from 2000 to 2022. No additional parameters were applied. The *PubMed* results were sorted using its “Best Match” filter [43] and the first 500 articles were retrieved. The *CINAHL Plus* results were sorted using its “Relevance” filter and the first 250 articles were retrieved. All 13 articles from *ERIC* were retrieved. Screening was conducted using *Covidence* software [44]. To promote transparency and reproducible methods, the detailed search strategy for *PubMed* is included as Supplementary File S1.

### Screening

**FIGURE 1:**
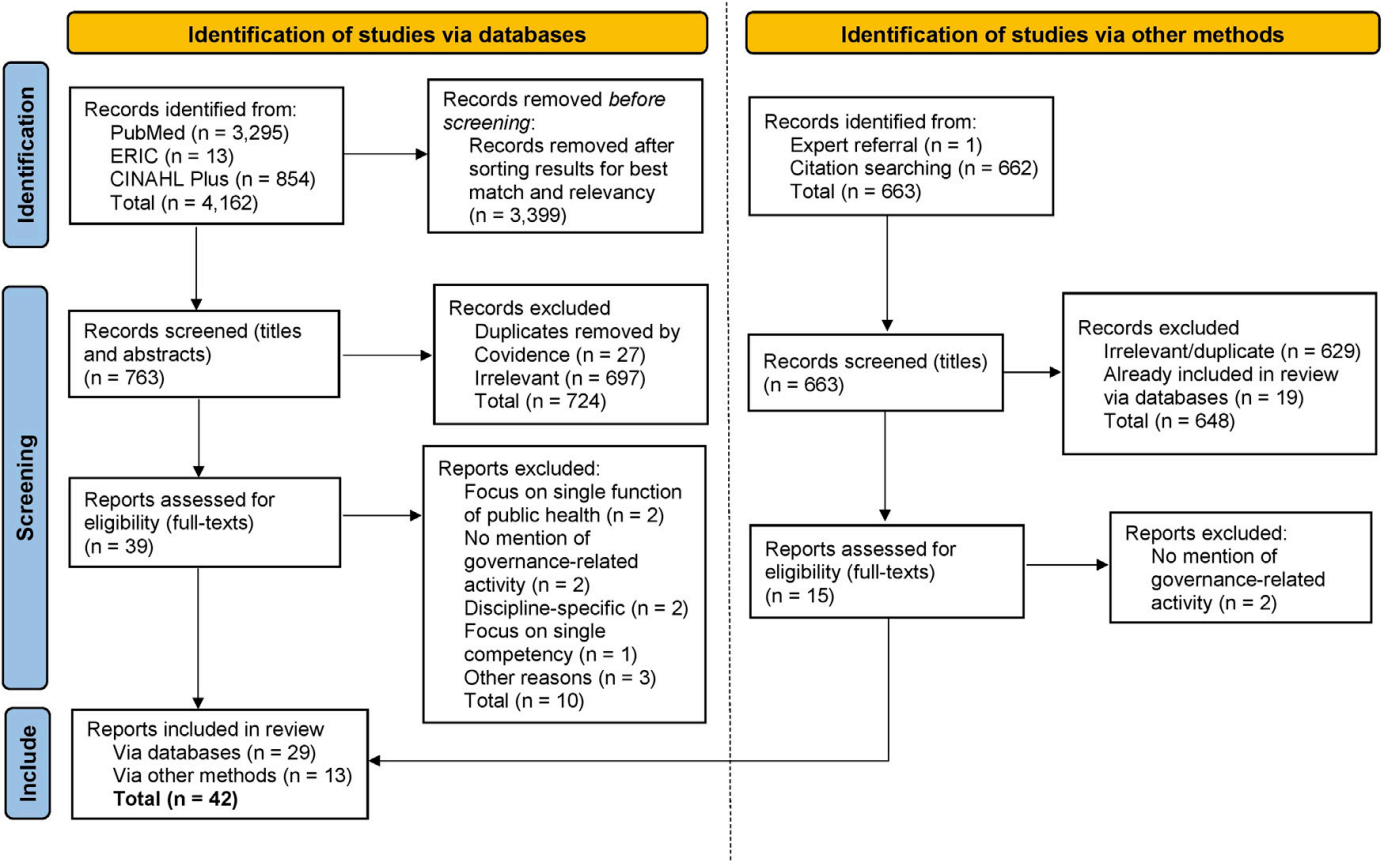
The Preferred Reporting Items for Systematic Reviews and Meta-Analyses (PRISMA) 2020 flow diagram depicting the article identification and screening process—adapted from Page et al. [76] (Canada, 2023).

A total of 763 unique articles were identified (see Figure 1 for the Preferred Reporting Items for Systematic Reviews and Meta-Analyses [PRISMA] flow diagram). HS, with training and experience in scoping review methodology [45], conducted the screening. A general set of inclusion and exclusion criteria (Table 1) was used to screen the articles and 39 articles were identified for full-text review.

**TABLE 1:** Eligibility criteria used during the article screening process (Canada, 2023).

Inclusion	Exclusion
• Focus on individual-level competencies (could be in practice or academic setting)	• Focus on organizational-level competencies
	• Discipline-specific (e.g., epidemiology, nutrition)
• Core, essential, or foundational core competencies for public health (CCPH) across multiple disciplines and roles	• Role-specific (e.g., nurses, physicians)
	• Focus only on a sole competency (e.g., leadership) or public health function (e.g., emergency preparedness and response)
• Mention of governance-related activity for public health competencies (e.g., development, evaluation, administration)	• No mention of governance-related activities
	• Analysis of the *content* of CCPH only

The full-text review resulted in the exclusion of 10 articles with reasons noted in Figure 1. Three of these articles were excluded after further reflection: two of them focused on educational CCPH at the undergraduate level [46] and doctoral level [47], and therefore did not overlap with our conceptualization of *core* or baseline competencies. The third article discussed CCPH for mid-tier or manager-level public health professionals [48] and was excluded for similar reasons. Articles with a focus on Master of Public Health (MPH) competencies were, however, included as an MPH is considered a foundational public health degree. The citations of the remaining 29 articles were then hand-searched and screened to determine eligibility for full-text retrieval and review. This resulted in 13 additional articles being identified for inclusion, bringing the total number of included articles to 42.

### Data Extraction

An extraction table was used to collect information from each article regarding the first author, publication year, title, country/region, primary purpose, type of literature, and findings relevant to the research question. HS conducted the data extraction and compiled the results into a table (Supplementary File S2). Key characteristics of the included articles are presented as a quantitative summary (verified by VO). Qualitative themes linked to governance were developed by reviewing the relevant findings and identifying salient themes as well as gaps (verified by LT). These themes are reported as a narrative synthesis. In line with rapid review methodology, critical appraisal of the articles was not conducted.

## Results

### Quantitative Summary

The 42 included articles were a mix of academic literature (published in peer-reviewed journals) (*n* = 33) and grey literature (*n* = 9). Of these, nine articles published a full set of CCPH as their primary focus. More than half (*n* = 24) of the included articles originated from the USA. Other commonly seen settings were the UK (*n* = 6), Canada (*n* = 4), Australia (*n* = 2), and India (*n* = 2). References (either as the focus of the article or in passing) were made to various CCPH sets published by the Council on Linkages Between Academia and Public Health Practice (COL) in the USA (*n* = 25 articles), PHAC (*n* = 9), Association of Schools of Public Health (ASPH) in the USA (*n* = 7), Association of Schools of Public Health in the European Region (ASPHER) (*n* = 6), Public Health England (PHE) in the UK (*n* = 6), Council of Academic Public Health Institutions Australasia (CAPHIA) (*n* = 5), and Public Health Association of New Zealand (PHANZ) (*n* = 2).

### Qualitative Narrative Synthesis

#### Theme 1: Collaboration and Partnership Across Multiple Actors is a Key Part of Developing CCPH

Many different types of organizations have led the development of CCPH. These include governmental public health agencies [2,11], membership-based and practice-focused public health associations [8], independent researchers [49,50], as well as academic associations and institutions [51–54]. Partnership, collaboration, and community engagement are key functions of governance [14,15] and were prominent across the articles. Networks of actors from academia, public health practice, and government coming together to establish sets of CCPH was commonly reported. The exact mix of actors involved may vary from setting to setting. For instance, CAPHIA collaborated with the Public Health Indigenous Leadership in Education group to reflect the importance of Aboriginal and Torres Strait Islander health in their CCPH [10] and PHANZ developed its CCPH in partnership with Māori Community Health Workers [8].

One of the most commonly used techniques to facilitate the collaborative development of CCPH is the Delphi method [49]. The Delphi method is consensus-oriented, involves input from key informants, and provides multiple opportunities for feedback and revision [49]. Transparency is a key part of the Delphi method as feedback from rounds of revisions is shared back with key informants to facilitate consensus in the subsequent rounds [49]. Furthermore, there are several cases where a broader consultative process occurred to collect feedback on draft CCPH from the general public health community, sometimes through open surveys as seen as in the PHAC and COL processes [2,12,55]. These are some examples of how the governance-related principles of inclusion, transparency, equity, and collaboration were reflected in the development of some CCPH sets.

#### Theme 2: Different Approaches to Reviewing and Revising CCPH

Continuous improvement is a key function of governance [14,15] and sustained efforts to ensure implementation and relevancy of CCPH is important. Most articles concurred that there is a need to periodically review and update competencies. However, there are a variety of approaches taken to address this. The PHANZ-CCPH for instance notes the importance of a regular review cycle but does not describe how the process should occur [8]. Alternatively, COL has a system whereby their CCPH are reviewed every 3 years and a decision is made to either continue as-is or revise [12]. The COL’s practices align with Calhoun et al.’s proposition that CCPH have a lifespan of three to 5 years [56] and Sharma et al.’s recommendation that CCPH should be reviewed every 3 years [50]. These recommendations come from the belief that CCPH reflect sociopolitical and cultural contexts as well as the diverse needs of a population, at a single point in time [5].

Other sets of CCPH have also gone through periodic updates and revisions (e.g., PHE and CAPHIA) [10, 11], albeit with relatively less consistency in their processes. While continuous improvement and sustainability are reflected in their practices, there is less explicit mention of an exact frequency and timeline for review and revision. Finally, there are some sets of CCPH such as those from PHANZ and PHAC that have not seen any new versions published since their initial release in 2007 and 2008, respectively [2, 8]. These cases stand out as most of the other established sets of CCPH have seen multiple iterations published.

#### Theme 3: Gaps in Monitoring, Implementation, and Resource Management of CCPH

Sets of CCPH have been used to support a variety of activities aimed at advancing public health practice and academia. Several articles from the USA involved analyses where sets of CCPH were used to capture workforce development needs and assess perceived changes in competency before and after a training program [57–64]. Other articles, including a few from Canada, focused on settings where sets of CCPH were used to review academic curricula [30, 50, 54] and assess students’ change in competence after a course, practicum, or program [65–67]. Furthermore, actors who were either developing new sets of CCPH for different regions or revising previous versions of CCPH, frequently consulted and referred to existing sets of CCPH internationally [49, 50, 53, 68, 69].

As CCPH can be applied in many contexts and by different actors, implementation, monitoring, and continuous improvement activities [14,15] may be difficult to coordinate. Of the included articles, there was only one instance where the lead developer of a set of CCPH conducted and published a formal review of its utilization and impact (PHE) [69]. Other evaluations and applications of sets of CCPH often came from researchers and analysts embedded in public health academic and/or practice settings. The monitoring of CCPH for utilization and impact should be a priority as a significant amount of financial, human, and volunteer resources go into the development, review, and revision of CCPH [70].

Lastly, there were some gaps in the findings related to aspects of resource management as a part of governance. Funding enables the delivery of CCPH-related activities, but details of what financial and human resources have been mobilized in the development, review, and revision of sets of CCPH were seldom described in the included articles. In some cases, the funding sources for CCPH-related development and revision activities are named (e.g., the Centers for Disease Control and Prevention for the COL-CCPH and ASPH-CCPH) [12, 56, 70]. However, details on the exact amounts and ongoing resource management were not discussed in the included articles.

## Conclusion

This rapid review explored what is known and not known about CCPH governance. There is substantial academic and grey literature on CCPH emerging from high-income countries and regions such as the USA, Canada, European region, Australasia, and the UK. Evidence from low- and middle-income countries was limited. The findings identified several examples of collaboration and community engagement in governance as well as different ways of approaching CCPH review and revision. However, there were minimal findings related to how sets of CCPH are monitored for implementation and impact as well as their overall resource management.

Public health activities involve collaboration between an expansive list of actors (e.g., professional associations, governmental departments, universities, and community-based organizations) [1] and this is reflected in the governance of CCPH. For instance, using the Delphi method to develop CCPH emphasizes aspects of collaborative governance such as consensus-oriented decision-making, inclusion of experts and community members, and transparency in processes [16, 20, 71].

Other aspects of governance such as continuous improvement and responsiveness are reflected in the ways that sets of CCPH are reviewed and updated. There is a strong case for periodic revisions of CCPH so that they can match evolving contexts and population needs. For example, climate change and antibiotic resistance are increasingly becoming the focus of public health work moving forward [72]. While a review cycle of 3–5 years has been proposed [50, 56] and implemented by COL [12], the ability to conduct reviews and revisions may depend on a variety of factors such as political climates, existing priorities, available resources, and capacity for implementation and monitoring. The gaps in monitoring and implementation could benefit from the application of a systems thinking perspective where activities and actors are connected within workforce and academic settings, and the aim is to improve public health system performance [4].

### Limitations

There are some limitations to note. The search was not exhaustive—three databases were accessed, a subset of identified articles was retrieved, and only articles published in English between 2000 and 2022 were considered. Furthermore, several included articles had a focus on MPH programs which are unlikely to be indicative of the broader public health workforce. In alignment with previous findings [73, 74], this study reaffirms that details on governance-related activities are seldom published. Batt et al.’s recent work is a step towards addressing this challenge by proposing guidelines for reporting competency framework development to better promote transparency and consistency [75]. Finally, both article selection and data extraction were conducted by one reviewer (HS). Considering these limitations and risks of selection bias, this study should be viewed as an analysis of the influential academic and grey literature on CCPH.

### Future Directions

The aim of this study was to capture considerations for how Canada should approach the governance of the PHAC-CCPH moving forward. The findings suggest that there should be systems, structures, and processes that support ongoing reviews, revisions, and monitoring of CCPH. Moreover, CCPH-related activities should be embedded within systems of workforce development and academic training that are linked to individual and organizational performance. Finally, governance activities should be connected to relevant governance functions [14, 15] and aim to advance the principles of good governance where possible [16–26].

Further work that builds on our findings and accounts for the limitations of this study needs to occur before more definitive lessons and recommendations can be drawn. Our next step is to conduct in-depth case studies of the CCPH published by PHAC, COL, PHE, PHANZ, CAPHIA, and ASPHER, where governance can be explored in more detail through targeted grey literature searches and consultations with key informants.

## Abbreviations

ASPHER, Association of Schools of Public Health in the European Region; ASPH, Association of Schools of Public Health in the United States of America; CAPHIA, Council of Academic Public Health Institutions Australasia; CCPH, Core Competencies for Public Health; COL, Council on Linkages Between Academia and Public Health Practice; NCC, National Collaborating Centre; PHAC, Public Health Agency of Canada; PHANZ, Public Health Association of New Zealand; PHE, Public Health England; UK, United Kingdom; USA, United States of America; WHO, World Health Organization.
