# Supplementary material for: The Governance of Core Competencies for Public Health: A Rapid Review of the Literature
Source: Public Health Rev. 2023 Sep 12;44:1606110. doi: 10.3389/phrs.2023.1606110 (PMC10520247; doi:10.3389/phrs.2023.1606110)
Supplement: Supplementary file 1 [file DataSheet2.docx]

**Supplementary File 2:** Summary of extracted results ordered by year of publication.

| First Author (Last Name) | Published (Year) | Title | Country/  Region | Primary Purpose | Type of Literature | Actual Set of Competencies | Relevant Findings |
| --- | --- | --- | --- | --- | --- | --- | --- |
| Day | 2002 | Core Competencies for Public Health Professionals: The New Look in Public Health Practice | United States of America (USA) | To provide an overview of the competency framework development process | Grey | No | - This article very briefly summarizes the development process for the first competency set developed by the Council on Linkages Between Academia and Public Health Practice (COL). - The development process involved a literature review, crosswalk analyses, review of the 10 Essential Public Health Services, and then drafting of an initial set of competencies. - A public comment period followed before the list was further refined and the final set published in 2001. - The COL core competencies for public health (CCPH) were intended for use in curriculum review and development, workforce training needs assessment, informing the development of discipline-specific competencies, and performance measurement. - The COL-CCPH built on a decade of work and engaged over 1,000 public health professionals during the public comment period. - Mentions funding from the Health Resources and Services Administration (HRSA) agency and staffing from the Public Health Foundation (PHF). |
| Mayer | 2003 | Are the Public Health Workforce Competencies Predictive of Essential Service Performance? A Test at a Large Metropolitan Local Health Department | St. Louis County, USA | To study the association between competencies and job performance using survey data from a local health department workforce | Academic | No | - This article was a quantitative cross-sectional study. - This study’s survey instrument relied on a preliminary version of the COL competencies available in 1999 and its analytic, cultural, program development, and communication domains. - The competencies could explain 2-20% of the variance in essential service performance after adjusting for career demographics. - The competencies have the potential to inform the design and content of public health workforce training. - The Missouri Department of Health, HRSA, and Centers for Disease Control and Prevention (CDC) supported this project. |
| Bartee | 2003 | Assessing Competencies of the Public Health Workforce in a Frontier State | Frontier state, Rocky Mountain region, USA | To “determine the level of perceived proficiency of a public health workforce based on the Public Health Practice Core Competencies" (p459) | Academic | No | - Used the COL-CCPH to develop and implement a survey of perceived proficiency of a public health workforce (as a needs assessment). - The survey was sent to public health employees representing areas in public health nursing, environmental health, public health, mental health, and management/administration. - A total of 401 responses were analyzed. Perceived proficiency varied by discipline with each group showing different strengths and weaknesses. These results can provide direction for continuing education/training opportunities. - This study was funded through a cooperative agreement with the University of Washington, School of Public Health, Northwest Center for Public Health Practice, Seattle, WA. |
| Van der Putten | 2006 | Assessing the Required Skill Mastery in Public Health Competencies in Thailand | Thailand | To understand which public health competencies are most relevant in Thailand | Academic | No | - This quantitative cross-sectional study utilized the COL-CCPH. Part of this research was identifying which of the competencies were most relevant to the public health training and practice setting in Thailand, according to the beliefs of the participants. - Survey respondents included 228 individuals representing academics, public health professionals, public administrators, and Ministry of Public Health staff. - The authors note that they drew the competencies from the COL-CCPH for this study but could have also looked elsewhere (e.g., World Health Organization [WHO]) to see what other CCPH existed. - This work helped inform a broader needs assessment of a graduate public health training program in Thailand. - A grant from the China Medical Board of New York supported this work. |
| Association of Schools of Public Health (ASPH) Education Committee | 2006 | Master's Degree in Public Health Core Competency Development Project Version 2.3 | North America  USA | To describe the set of CCPH intended to serve as “a resource and guide for those interested in improving quality and accountability of public health education and training” (p3) | Grey | Yes | - A national effort to develop competencies was undertaken between August 2004 and August 2006 (two years) that involved over 332 members of academic and practice community. - In phase one, the ASPH Education Committee established six working groups (consisting of a mix of academic and practice representatives) corresponding to six areas of public health (i.e., biostatistics, environmental health sciences, epidemiology, health policy and management, social and behavioural sciences, and public health biology). - Each working group was asked to develop the top 8-10 discipline-specific competencies required by an average Master of Public Health (MPH) student via a modified Delphi method (three rounds of surveys and expert panel discussions). - The working groups published their progress throughout the process and an email address was provided to track input from members and the broader public health community. - Another working group was formed from the chairpersons of the initial six working groups and charged with integrating the competencies into a single cross-cutting set. An initial version of the competencies was published, and stakeholders were invited to review. This process was repeated a few times with iterations of the competencies being published continuously. - In phase two, six new working groups were formed to represent the emerging cross-cutting competencies (i.e., communication, diversity and cultural proficiency, leadership, professionalism and ethics, program planning and assessment, and systems thinking). Another round of Delphi and expert panel discussion was conducted. The next step involved the integration of both the area-specific and cross-cutting competencies into a single model. |
| Oppewal | 2006 | Comparison of the Dissemination and Implementation of Standardized Public Health Nursing Competencies in Academic and Practice Settings | USA | To assess the use of the COL core competencies for public health professionals by public health nurses | Academic | No | - A total of 334 individuals participated in this quantitative cross-sectional study. Nursing faculty/academic professionals are adopting and using the COL competencies at a much faster rate than nursing practitioners. - This research aimed to study the diffusion and uptake of the COL-CCPH and the competencies released by the Quad Council of Public Health Nursing Organizations. The Rogers (1995) diffusion of innovation framework was referenced. - Some background information about the COL-CCPH was also shared (e.g., the original development process spanned 10 years and leaders from national organizations in public health academia and practice led the development effort with funding provided by the CDC and HRSA). - A reference is also made to how the Institute of Medicine report, the Future of Public Health (1988), initially highlighted the need for core public health competencies and how this influenced future work. - The researchers noted that as significant financial, personnel, and volunteer resources went in to establishing a set of CCPH, it is important to study whether and how the core competencies are being used and examine their impact. |
| Public Health Association of New Zealand (PHANZ) | 2007 | Generic Competencies for Public Health in Aotearoa-New Zealand | New Zealand | This is the generic set of competencies for public health in the context of New Zealand | Grey | Yes | - This set of CCPH was published by PHANZ in association with: (1) Health Promotion Forum of New Zealand; (2) Māori Community Health Workers; (3) New Zealand Institute of Environmental Health; and (4) Public Health Nurses Section of New Zealand Nurses Organization. The Australasian Faculty of Public Health Medicine also assisted during the development process. - The document makes specific reference to Te Tiriti o Waitangi and active participation of Māori as well as protection and improvement of Māori health status. - This CCPH set was developed as a part of the Public Health Workforce Development Plan. The document notes that the competencies "will be reviewed at regular intervals and updated to reflect changes in public health practice” (p12). |
| Public Health Agency of Canada (PHAC) | 2008 | Core Competencies for Public Health in Canada Release 1.0 | Canada | This is the competency framework which describes the core competencies for public health practice in Canada | Grey | Yes | - A reference is made to the Building the Public Health Workforce for the 21st Century report (2005) and how it proposed identifying core competencies as a key step towards strengthening public health capacity. - In 2005, the Joint Task Group on Public Health Human Resources developed a draft set of competencies and recommended that PHAC undertake a process to further develop the CCPH set. - Preliminary consultation and discussion organized by PHAC with public health stakeholders across the country led to a second draft and complementary glossary being developed in 2006. - This was then used to consult with a large number of practitioners and representatives from all levels of government through regional meetings across Canada, a national survey, implementation pilots, and engagement with specific discipline and professional organizations. Following this process, the final set of competencies was released in 2008. |
| Calhoun | 2008 | Development of a Core Competency Model for the Master of Public Health Degree | USA | To provide an overview of the development process of the ASPH core competencies for the MPH degree in the USA | Academic | No | - The ASPH began development of MPH core competencies in 2004 with support from CDC. These CCPH were developed over the span of two years (2004-2006) under the guidance of the ASPH Education Committee in two phases. - Phase one involved discipline-specific competencies (with working groups related to the domains of biostatistics, environmental health sciences, epidemiology, health policy and management, social and behavioural sciences, and public health biology). Phase two involved cross-cutting competencies (with working groups related to domains of communication, diversity and cultural proficiency, leadership, professionalism and ethics, program planning and assessment, and systems thinking). Working groups comprised of individual experts drawn from member universities and selected leaders from practitioner organizations developed the initial competencies with consensus, led by a working group chair or co-chairs. - A modified Delphi process and surveys (three rounds) were used to further refine competencies. Iterations of the competencies and development process were published to maintain open communication with ASPH members and the public health community (including establishing an email address where community members could provide input). - A total of 135 members participated in phase one and a total of 197 members participated in phase two. In total, more than 400 individuals provided their input on the development of the final ASPH core competencies. - These competencies are intended to "serve as a resource and guide for those interested in improving the quality and accountability of public health education and training" (p1606). - The authors mention how competency sets generally have a lifespan of three to five years and thus further refinement and updating based on new thinking and future challenges is necessary. |
| Moser | 2008 | Core Academic Competencies for Master of Public Health Students: One Health Department Practitioner's Perspective | USA | To comment and reflect on the development process of the ASPH MPH core competencies | Academic | No | - The author notes that the ASPH core competencies are intended to be a work in progress and should be updated on an ongoing basis. - "There were 37 practitioners among the 332 participants in the core and resource groups assembled for the development of the ASPH MPH core competencies" (p1560). The author states that in the future, more practitioners should be involved in the development process. |
| Edgar | 2009 | Construct Validity of the Core Competencies for Public Health Professionals | Missouri, USA | To investigate the construct validity and reliability of the Council's core competencies for public health professionals | Academic | No | - The authors briefly mention how the development process for the competencies involved the gathering of comments and feedback from public health professionals through a public comment period, focus groups, and conference sessions. - Assessing the validity and reliability of any CCPH is important. The authors believe that the development process involved evidence of face and content validity but construct validity and reliability are still lacking. - A total of 566 individuals completed the survey to self-report their proficiency in the COL competencies, slightly modified for this quantitative cross-sectional study. - The principal component analysis showed the strong construct validity of this set of competencies, but the authors note that additional studies will need to be conducted to study their reliability. |
| Genat | 2010 | New competencies for public health graduates: a useful tool for course design | Australia | To provide an overview and context for the Australian Network of Academic Public Health Institutions (ANAPHI) Foundation Competencies for Master of Public Health Graduates in Australia | Academic | No | - In 2006, ANAPHI (which eventually became known as Council of Academic Public Health Institutions Australasia [CAPHIA]) and the federal Department of Health and Ageing's Public Health Education and Research Program held a workshop to discuss public health competencies that could be expected from an MPH graduate. - From 2006 to 2007, the program led a national inquiry based on an online Delphi process to further refine the MPH competences discussed previously. Participation in this process included the attendees of the initial workshop, public health academics, employers from public sector organizations, and policymakers. - After a hiatus, an official set of competencies for MPH graduates was published in 2009. The competencies, called the Foundation Competencies for Master of Public Health Graduates in Australia, are aligned with the competencies set at a master’s level within the Australian Qualifications Framework. - This set of CCPH is considered to be the minimal/baseline set of competencies expected of any MPH graduate from a university in Australia and can be used to inform curriculum development. - The authors make a passing reference to the CCPH established by PHAC, COL, and ASPHER. |
| Stewart | 2010 | Public Health Workforce Training: Application of the Council on Linkages' Core Competencies | Arkansas, USA | To study the change in competency levels after the implementation of a workforce development program in a state public health department | Academic | No | - This quantitative research incorporated the COL core competencies into a workforce development program at the Arkansas Department of Health. - The researchers saw the COL-CCPH as an effective tool for both informing a workforce/professional development program as well as in evaluating training outcomes. - The National Association of County and City Health Officials and the Association of State and Territorial Health Officials have encouraged using the COL-CCPH in public health workforce development and assessment. - The survey used in this study was based directly on the COL competency statements and participants were asked to self-report their proficiency. The study showed gains in competencies following the training program. |
| Birt (the Association of Schools of Public Health in the European Region [ASPHER]) | 2011 | European Core Competencies for MPH Education (ECCMPHE) | Europe | This document is ASPHER's set of public health competencies to be achieved by MPH graduates | Grey | Yes | - ASPHER began its competencies development work in 2006. The development process of the original set of ASPHER competencies included the involvement of ASPHER member organizations, conferences, and participation from European ministries of health and public health employers. - Using its initial CCPH set, ASPHER developed the ECCMPHE for a more specific purpose of informing MPH educational and curriculum development. - An acknowledgement is provided to The Executive Agency for Health and Consumers (EAHC) Operating Grant number 2010 32 02; Nordea Danmark Fonden; University of Aarhus, Aarhus, Denmark; The Central Denmark Region; and Ecole des Haute Etudes de la Santé Publique (EHESP), Paris, France, for financially supporting this work. |
| Birt | 2011 | The Developing Role of Systems of Competences in Public Health Education and Practice | USA and UK | To review the achievements and failings of the USA and UK public health competencies initiatives | Academic | No | - Sustainability of CCPH sets require systems and structures to support continuous review and updating. Competencies should reflect the local, regional, and national contexts and needs at a given point in time. - The development process of three CCPH sets is discussed: (1) ASPH; (2) Faculty of Public Health in the UK; and (3) ASPHER. The ASPH process is well described by Calhoun (2008) and others. - The UK Faculty of Public Health’s competencies established in the 1990s began as a means of measuring "the extent to which each trainee had demonstrated a proper grasp of [certain] knowledge and skills" (p139). Eventually, four UK government health departments financed the development of a new set of competencies called the PHKSF. The UK development process appeared to be centrally managed and coordinated in a top-down manner. The UK competencies were designed to serve both the needs of educational and workplace settings. ASPHER's competencies were initially designed to inform public health education. Member schools of ASPHER were invited to participate in six working groups in which approximately 100 professors and researchers participated. Five of the working groups addressed primary public health domains (i.e., methods in public health; social environment; physical, chemical and biological environment; health policy, organization, management and economics; and health promotion). The sixth group worked on cross-cutting themes (i.e., strategy development, ethics). An initial set was published, and all European ministries of health were invited to a conference to discuss the competencies with ASPHER representatives. A total of 27 countries participated in the conference and the competencies were revised. The development appeared to be bottom-up as ASPHER facilitated the process with lots of opportunities for input from educational, governmental, and workforce representatives. ASPHER has further delineated their sets of competencies to include a general list for professionals as well as a list to inform MPH education. ASPHER also had planned on developing a list to advise general public health practice. |
| Hawley | 2011 | Competency-Based Impact of a Statewide Public Health Leadership Training Program | Kansas, USA | To evaluate and assess the effectiveness of a public health leadership institute training program | Academic | No | - This quantitative study used the COL-CCPH which the authors see as a tool for assessing skill level within a workforce. - The state of Kansas has developed a public health leadership institute to assist with advancing competencies within individuals in the workforce. - A total of 109 individuals who participated in the leadership program were invited to participate in an evaluation. Online surveys were administered pre- and post-training asking the respondents to self-report their skill level on the COL core competencies (*n* = 74 completed surveys for this portion). - Participants reported significant improvements in all core competency domains post-training (*p* < 0.001). - The authors acknowledged the financial support of the Kansas Health Foundation towards the Kansas Public Health Leadership Institute. |
| Wells | 2012 | Assessing Integration of Clinical and Public Health Skills in Preventative Medicine Residencies: Using Competency Mapping | Michigan, USA | To map the learning objectives from a community health center rotation for medical students to clinical preventative medicine competencies and the COL core competency framework | Academic | No | - This study focused on the potential for preventative medicine residencies to advance public health skills and training and medical school students. - The Accreditation Council for Graduate Medical Education's Preventative Medicine Residency Review Committee (ACGME-RRC) has specified competencies for preventative medicine residents. - This research reviewed documents to map a medical school educational component (i.e., a community health center rotation within a residency program) to both the ACGME-RRC competency framework and a subset of the COL tier two core competencies (2010 version). - The authors note that a competency mapping exercise could be useful in assessing and informing curriculum and training programs. - HRSA was acknowledged for providing support to the University of Michigan Preventative Medicine Residency. |
| Sharma | 2013 | Designing the Framework for Competency-Based Master of Public Health Programs in India | India | To develop a national MPH competency framework in India | Academic | Yes | - This quantitative research aimed to define the competencies for MPH programs in India in order to standardize and guide curricula development across the country. - Four steps were taken. First, a situational assessment was completed whereby MPH programs were identified through various methods and information was collected through a standardized questionnaire. Second, a survey of public health professionals was conducted to gather input on the current competencies and curriculum of MPH programs in India. A total of 122 individuals completed the survey and represented stakeholders from academia, public health practice, national and international organizations, NGOs, industry and civil society. A national consultation was held next to discuss the results of the previous steps. This involved actors from MPH-offering academic institutions, the Ministry of Health and Family Welfare - Government of India, Medical Council of India, National Board of Examinations, University Grants Commission, WHO, Public Health Foundation of India, and more. The consultation revealed a need to also review existing competency-based education frameworks for MPH programs to assess if and how they could be adapted to the Indian context. Finally, these activities culminated in the development of a framework which identified core public health functions in India and associated learning outcomes. - The authors noted that Australia, USA, Europe, and Canada had spent time and expertise in developing CCPH to support workforce training. They believed that reviewing these sets and their development could inform the work conducted in India. - The following sets of CCPH were reviewed and consulted: (1) ANAPHI; (2) COL; (3) ASPH; (4) ASPHER; and (5) PHAC. - The authors, referencing other documents, note that the ASPHER competencies were driven in part by the Bologna Declaration/Process which aimed to advance international cooperation and academic exchange. - The authors note that the development of a set of CCPH is an iterative process and as the needs and demands of the public health system evolve, competencies must be reviewed and redefined periodically (e.g., every three years). |
| COL | 2014 | Core Competencies for Public Health Professionals | USA | This document is the COL core competency framework published in 2014 | Grey | Yes | - The COL is a collaborative of 20 national organizations that aim to improve public health education, practice, and research. The 20 organizations are listed in detail in the document. - The COL is funded by the CDC and staff support is provided by the PHF. - The 2014 set of CCPH represents the culmination of more than two decades of work, which first started with the development of universal competencies in 1991 by the Public Health Faculty/Agency Forum. Work began to transition towards core competencies in 1998 and involved the input of professionals across the country, a workgroup, and a public comment period with over 1,000 comments. This resulted in the development of the first set of CCPH in 2001. - The COL committed to revisiting their set of CCPH every three years to determine whether a revision was needed. The competencies were reviewed in 2004 and it was determined no updates were required. The 2007 review suggested a revision and the COL initiated a similar process as before with a workgroup and over 800 comments from professionals. This revised set, released in 2010, added tiers to the competencies. The next review, in 2013, suggested another revision. Thus, the 2014 set was developed using a similar process as before. - A set of complementary resources and tools are also made available to assist in the use of the framework. An email address is also provided in the document for ongoing feedback and suggestions. |
| Britten | 2014 | Using core competencies to build an evaluative framework: outcome assessment of the University of Guelph Master of Public Health program | Ontario, Canada | To determine whether MPH graduates are receiving adequate training using the PHAC competencies as an evaluative framework and identifying areas for improvement | Academic | No | - This quantitative research involved surveys (including retrospective and qualitative questions) that showed MPH graduates had gained proficiency in the core competencies through their core and elective courses and practicums. - The competencies can be used as an informative outcome assessment tool. Thirty-five students completed the survey. Students were asked about how program experiences (courses, practicums, experiences with faculty members) impacted their proficiency in the core competencies. A series of retrospective pre- and post-program competency assessments were also included in the survey. The core competencies could be used as an evaluative framework to measure an MPH program's effectiveness in helping graduates achieve proficiency. |
| Polivka | 2014 | Congruence Between Position Descriptions for Public Health Nursing Directors and Supervisors With National Professional Standards and Competencies | Ohio, USA | To analyze how well job descriptions for public health nursing directors and supervisors incorporate standards of public health nursing and competencies for managers | Academic | No | - The researchers used competencies from the COL and the 2007 American Nursing Association Public Health Nursing Scope and Standards of Practice competencies as analytical tools. - A total of 94 position descriptions were analyzed. Overall, position descriptions addressed an average of 44% of the Council's competency domains. - This research was funded by a Quick Strike Research Fund from the Public Health Practice Based Research Network National Coordinating Center, University of Arkansas, and Robert Wood Johnson Foundation. |
| Neiworth | 2014 | Charting a Course to Competency: An Approach to Mapping Public Health Core Competencies to Existing Trainings | Kansas, USA | To describe the process by which this research team mapped public health competency frameworks to learning objectives and assessments of existing training programs | Academic | No | - State and local public health departments are increasingly using competency frameworks for workforce development and assessment. - These researchers chose the COL competencies as well as two other preparedness and response-focused competency frameworks as mapping tools. - These frameworks were applied to 62 online courses available to the public health workforce to advance their training. The mapping process is outlined, but the results of the analysis are not presented. - This work was supported by the United States Department of Health and Human Services HRSA through a cooperative agreement with the Rollins School of Public Health, Emory University. |
| Zwanikken | 2014 | Validation of public health competencies and impact variables for low- and middle-income countries | Low- and middle-income countries (LMICs), global | To design a set of public health competencies using the learning objectives of six different MPH programs offered in LMICs using the COL framework as a reference | Academic | Yes | - The resulting competency framework differed from the COL in its scope and focus on social determinants of health, context specificity, and inter-sectoral competencies. - A modified Delphi method used in this study included input from experts and MPH alumni from China, Vietnam, South Africa, Sudan, Mexico, and the Netherlands. Initially, representatives from six MPH programs met and developed an initial consensus on a set of public health competencies. This was followed by two rounds of Delphi: first with the public health experts (*n* = 31) and then with the MPH alumni (*n* = 30). An attempt was made to incorporate input from participants with various professional, gender, and cohort background. - The resulting competency framework could be used to design and evaluate MPH programs, assess individuals and teams, and support professional development in LMICs. - In the study's literature review, the authors mention the development of a variety of core competency frameworks by: (1) COL; (2) PHAC; (3) ASPHER; (4) the Public Health Skills and Career Framework in the United Kingdom (UK); (5) ANAPHI. Referencing other literature, the authors note that these frameworks were mostly developed through group discussions and/or modified Delphi methods with various degrees of input from academia and public health practitioners at multiple levels. - This study was funded by the respective institutions of the authors and by the Ministry of Foreign Affairs of the Netherlands. |
| Zahner | 2014 | Public Health Practice Competency Improvement Among Nurses | Wisconsin, USA | To assess the potential change in competency for nurses enrolled in a public health training project | Academic | No | - The quantitative survey assessment used in this pre-post study was based on another tool specific for public health nurses and not a core competency framework. - Participation in the workforce development program led to improvements in competencies for some domains. - The authors make a reference to the COL core competency framework and mention how nursing education programs have been influenced by it (referencing other citations). - Again, referencing other citations, they also mention how competency assessment tools related to the COL framework have been used to guide academic education and workforce development in general. - Publication of this article was financially supported by the CDC through a cooperative agreement with the PHF and the University of Michigan’s Center of Excellence in Public Health Workforce Studies. The research itself was supported by funding from the HRSA and the University of Wisconsin-Madison, School of Nursing, Research Committee Faculty Research Fund. |
| Public Health England (PHE) | 2015 | Review of the Public Health Skills and Knowledge Framework (PHSKF) Report on a series of consultations | UK | To review and evaluate the 2008 PHSKF | Grey | No | - This report focused on the review of the UK's 2008 PHSKF. This review process involved consultation through local workshops across the UK (180 participants), an online survey including qualitative components (520 respondents) and a trial of an online e-portfolio (100 participants). - Workforce from all levels, sectors, and four UK nations were engaged in the process. The review process intended to explore the current use and application of the competency framework and obtain feedback from the frontline workforce on its strengths, weaknesses, and how it could be updated. - Prior to the consultation, a literature review of other frameworks was also conducted (which involved the PHAC and COL frameworks). The PHSKF originally launched as the Public Health Skills and Career Framework in 2008. It served as a resource that described the skills and knowledge needed within all the domains and levels of the UK public health workforce. - The development of the 2008 framework was informed by a variety of strategic documents (e.g., National Occupational Standards, Faculty of Public Health's curriculum, National Health Services Knowledge and Skills Framework). - In 2012, a national workforce advisory group commissioned a review that resulted in the recommendation to change the Knowledge and Careers framework to the Skills and Knowledge Framework. |
| Harrison | 2015 | The effect of using different competence frameworks to audit the content of a masters program in public health | UK, global | To conduct an audit of the existing MPH curriculum and map how well it addresses common MPH competency frameworks | Academic | No | - Four competency frameworks (ASPH; ECCMPHE; Faculty of Public Health Part A Exam, UK; Specialist Training Program, UK) were mapped to an MPH curriculum. - "Using different competence frameworks to audit the curriculum of an MPH program can give different indications of its quality" (p1). The review helped highlight potential gaps in the content of this one MPH program. - No external funding was received for this research. |
| Wallar | 2015 | Collaborating, Competencies and the Classroom: A Public Health Approach | Ontario, Canada | To assess the change in competency of MPH students after having completed a capstone business plan assignment in collaboration with local public health professionals | Academic | No | - This research utilized the PHAC core competency framework to assess proficiency among MPH students using quantitative (pre-post surveys) and qualitative (focus group) methods. - The authors see this competency framework as being relevant to informing MPH program curricula. They also serve as a self-assessment tool to be used in workforce development. - This was research was conducted at the University of Guelph. Students advanced proficiency in competencies through this particular assignment. - The researchers note that a limitation of using the PHAC framework is that the competency statements were written for public health professionals rather than graduate students. Therefore, they recommended the development of MPH-specific competency frameworks for the Canadian context. - Funding for this research was provided by Open Learning and Educational Support at the University of Guelph. |
| CAPHIA | 2016 | Foundation Competences for Public Health Graduates in Australia 2nd Edition | Australia | This document contains a comprehensive set of competencies to benchmark curriculum development of MPH and undergraduate programs in public health in Australia | Grey | Yes | - Funding for the updating of these competencies was provided through a Public Health Education and Research Program administered by the federal Department of Health and Ageing. - The original competencies were developed through contributions from academic, community organization, and public organization representatives. This edition of the competencies was developed by CAPHIA with input from all 23 universities offering MPH programs in Australia in 2015. The review process was three years long and led by three main editors drawn from universities represented in CAPHIA. - Input from the Public Health Indigenous Leadership in Education group was also sought and obtained to reflect the importance of Aboriginal and Torres Strait Islander health in broader public health policy and practice in Australia. - The Delphi process included semi-structured surveys, telephone follow-ups, and the reporting of interim findings to CAPHIA committees for further feedback and iterations. - These are a minimum or baseline set of competencies that can be expected from a typical public health student upon graduation. It also includes examples of discipline-specific competencies. The competencies are meant to be used in conjunction with the broader Australian Qualifications Framework. |
| PHE | 2016 | Public Health Skills and Knowledge Framework | UK | To describe the generic activities and functions undertaken by the public health workforce in the UK | Grey | Yes | - This framework was developed in collaboration with multiple agencies across the UK: (1) PHE; (2) Public Health Wales; (3) National Health Services Scotland; and (4) the Public Health Agency of Northern Ireland. - The Public Health Skills and Knowledge Framework (PHSKF) was first published in 2008 as the Public Health Skills and Career Framework. It was initially developed through the collaborative efforts of public health agencies across the UK. - In 2012, a partial refreshing and renaming of the framework took place. The review process was led by the four agencies listed previously and resulted in the updating of the framework. |
| Brown | 2017 | An Evaluation of a Competency-Based Public Health Training Program for Public Health Professionals in Kansas | Kansas, USA | To “evaluate the effectiveness of a piloted competency-based public health training program formed out of an academic-public health agency partnership" (p447) | Academic | No | - The COL competencies were used in a mixed methods needs assessment to identify training needs for public health staff and inform the development of a training program. - They were also used in surveys to capture the respondents’ level of confidence in applying acquired skills related to the competencies following the workshop. There were 72 staff that participated in this research. - Kirkpatrick’s (1994) four-level training evaluation framework was referenced. |
| Siemon | 2018 | Assessment of public health core competencies in prelicensure baccalaureate nursing students | Idaho, USA | To assess the level of public health competency among nursing students before and after completing community health courses | Academic | No | - This quantitative study utilized the COL-CCPH (2014) competency assessment tool for Tier 1 Public Health Professionals to measure competency advancement in an educational setting. - This tool aims to measure the self-reported level of competency within the COL competency framework. The authors, referencing American Nurses Association (2013), note how the COL core competencies were the basis for a separate set of discipline-specific competencies developed for nurses: the Quad Council Coalition of Public Health Nursing Organizations Core Competencies for Public Health Nurses. - This study found that students self-reported higher levels of competency after the courses compared to before them. The PHF assessment tool can be used as an evaluative method to assist nursing educators teaching public health nursing. |
| Shickle | 2019 | The applicability of the UK Public Health Skills and Knowledge Framework to the practitioner workforce: lessons for competency framework development | UK | To assess the utilization of competencies in the UK PHSKF among public health practitioners | Academic | No | - This was a qualitative study in which 15 group interviews were conducted with 51 public health practitioners from 8 local health authorities in the UK. The PHSKF was seen positively even though no participants had read or utilized the framework. Participants felt that the PHSKF would be a suitable resource for informing the development of practitioner apprenticeship training curricula. - The authors note that often, details for how public health competency frameworks were produced are not provided (other than references being made to consultations with various organizations and actors). - Of the participants who had seen the PHSKF, they preferred the newer and simpler layout. Further study of public health competency framework implementation is needed. |
| Grimm | 2019 | Creating Customized Workforce Development Plans for Medium-to-Large Public Health Departments | Nebraska, USA | To describe the process of a public health workforce assessment and demonstrate insights of the needs assessment | Academic | No | - This quantitative cross-sectional study adapted the COL core competencies into a training needs assessment survey, to which 128 individuals responded. - The authors noted that local health departments should continually reassess their workforce. - This study reported on the workforce development process from 2016-2019 (three years) and another article – Grimm et al. (2022) reports on the 2019-2022 plan. - The authors reported that they did not receive any financial support for this work. |
| Sathiakumar | 2019 | Building global partnerships through shared curricula for an MPH programme in India and Sri Lanka | India and Sri Lanka, Asia | To describe the design and implementation process of a competency-based MPH program | Academic | No | - This article describes the participatory process by which two locally-adapted and competency-based MPH curricula were developed for the Manipal Academy of Higher Education (India) and the University of Kelaniya (Sri Lanka). - The development of the curriculum and program was led by a 10-member working group comprised of senior faculty from three South Asian universities and one from the United Kingdom. The working group developed the curricula in one year through several meetings, discussions, and a review process. The chair of the working group and a research associate: (1) conducted in-country needs assessments pertaining to public health skills; (2) reviewed curricula from several Council on Education for Public Health (CEPH)-accredited schools in the USA, Aga Khan University (Pakistan), and others; and (3) completed a literature review of existing public health competency frameworks. - Three frameworks were identified as being relevant to informing the emerging MPH curricula: (1) ASPH; (2) WHO; and (3) CDC. - A brief description of the ASPH MPH competency framework development process is provided. Between 2004 and 2006, 332 representatives from the academic and practice communities of the ASPH Education Committee contributed to developing MPH competencies. Eventually, they were superseded by CEPH's MPH and DrPH Foundational Public Health Knowledge and MPH Foundational Competencies released in 2016. The ASPH competencies remain a relevant resource (cited Krisberg [2017]). - The WHO competencies refer to the framework led by WHO in collaboration with public health institution representatives from the Southeast Asia Region. This group developed a set of competencies for MPH programs in 2003. - Thirdly, the CDC competencies refer to a discipline-specific framework developed for epidemiologists. - The working group described in this study reviewed the three competency frameworks, conducted a crosswalk analysis, and then mapped them to courses. The competency frameworks played a key role in developing and designing the MPH curricula. - This work was supported by the University of Alabama at Birmingham International Training and Research in Environmental and Occupational Health program, from the National Institutes of Health – Fogarty International Center. |
| Bornioli | 2020 | Evaluation of the UK Public Health Skills and Knowledge Framework (PHSKF): implications for international competency frameworks | UK | To formally evaluate the PHSKF (2016 edition). The evaluation aimed to determine the impact of the revised framework and its utility | Academic | No | - This study was based on a sequential explanatory mixed methods design. - There were 298 participants in the surveys and 18 in the telephone interviews. Participants included those from all four UK nations and various sectors. - Most respondents had used the PHSKF rarely or occasionally. 87% of users found it useful and 82% thought it was easy to use. Common purposes for the use of the PHSKF included workforce and professional development (e.g., standard-setting, identifying training needs). 23% of respondents had never used the PHSKF (of which 47% did not know about it and 31% reported having no need to use it). - Public health competency frameworks are rarely, if ever, formally evaluated for their impact. A brief timeline of events is provided: in 2008, the Public Health Skills and Career Framework was developed by four national public health agencies and derived from the UK Faculty of Public Health curriculum. - PHE led a consultation on the initial framework in 2015 to simplify it and shift the focus from competencies to functions and capabilities. Respondents also reported uncertainty around the aims, scope (e.g., does it apply to all public health professionals and levels?), and audience of the PHSKF. - Some respondents preferred to use the UK Public Health Registrar standards (must be met to be included on a voluntary register of public health practitioners) as there were clearer career outcomes relative to the PHSKF. - A “system” and explicit “buy-in” to facilitate the use of PHSKF by individuals and organizational leaders appeared to be missing, and thus limited the potential use and impact of this framework. - This research was funded by PHE. |
| Coombe | 2020 | Practical competencies for public health education: a global analysis | Global | To map existing public health competency frameworks onto the elements of the World Federation of Public Health Associations' Global Charter for the Public's Health | Academic | No | - In this study, multiple competency frameworks (i.e., the ASPHER, CAPHIA, CEPH, PHAC, and PHANZ competencies) were mapped against the Global Charter for the Public's Health. - The authors noted that the way in which public health competencies were "developed, subsequent evolutions, and their intended uses, differ considerably and these histories are not necessarily included in the resulting documents" (p1160). - This analysis confirmed that each competency framework reflects the temporal, geographical, and political issues occurring within the context in which they were developed. The Aotearoa-New Zealand competency framework is unique in its focus on Māori health and Te Tiriti o Waitangi. |
| Matthews | 2021 | Can Public Health Workforce Competency and Capacity be built through an Agent-based Online, Personalized Intelligent Tutoring System? | USA | To investigate the capability of an agent-based, online personalized intelligent tutoring system to deliver public health workforce training | Academic | No | - This research referred to the COL core competencies for public health professionals. The tutoring program they developed addresses four out of the eight domains of the COL competency framework. Thus, the competencies were a way to understand which aspects were covered by this training program. |
| Apatu | 2021 | Where Are We Now? A Content Analysis of Canadian Master of Public Health Course Descriptions and the Public Health Agency of Canada's Core Competencies | Canada | To “examine the degree to which Master of Public Health (MPH) programs’ course descriptions align with the Public Health Agency of Canada’s (PHAC’s) core competency categories in order to identify strengths and training gaps in such programs across Canada" (p201) | Academic | No | - This qualitative study used the PHAC competencies to analyze MPH courses in Canada. A total of 209 courses were analyzed across 18 universities. This content analysis identified which competencies were best addressed by required courses in MPH programs (i.e., those related to public health sciences as well as assessment and analysis). It also identified that the less frequently addressed competencies were: (1) diversity and inclusiveness; (2) partnerships, collaboration, and advocacy; (3) communication; and (4) leadership. |
| Taylor | 2021 | Core Competency Gaps Among Governmental Public Health Employees With and Without a Formal Public Health Degree | USA | To examine the role of having a public health degree as it relates to core competencies among governmental public health employees | Academic | No | - This quantitative cross-sectional study is based on the 2017 Public Health Workforce Interests and Needs Survey and utilizes the 2014 COL competency framework. - The researchers saw the competency framework as an effective tool to identify and address skill gaps and training needs. - The Public Health Workforce and Interest Needs Survey is a "nationally representative survey of the US state and local governmental public health workers" (p21). - The COL competency framework is included in the survey to assess skill level and perceived importance. This analysis included 30,276 respondents. The researchers also referenced the 2016 CEPH accreditation criteria (which includes a competency framework) in their discussion. |
| Amos | 2022 | Developing Complex, Cross-Cutting Skills in the Public Health Workforce: Using a Crosswalk Analysis to Map Public Health Competencies to Strategic Skills for the Governmental Public Health Workforce | USA | To analyze three public health competency frameworks and depict their relationships between one another | Academic | No | - This study analyzes the 2014 COL competencies. The authors mentioned how another set of competencies, developed more broadly for a variety of professions to advance population health skills, was developed using the COL framework as a baseline. - The crosswalk analysis showed a considerable overlap between the COL competencies, PHF's Competencies for Population Health Professionals, and the Strategic Skills for the Governmental Public Health Workforce. In addition, each framework had its own way of expressing competencies and there were a few competencies unique to each framework. - The existence of multiple and overlapping public health competency frameworks may provide health departments with challenges of having to choose one set to implement in their workforce setting over another. - This project was supported by HRSA under a Regional Public Health Training Center Program for $767,470 [USD] (2021). |
| Grimm | 2022 | The Most Important Skills Required by Local Public Health Departments for Responding to Community Needs and Improving Health Outcomes | Nebraska, USA | To “determine the professional skills that are most important for local health departments to respond to large public health issues and challenges that are having a major impact on their communities" (p79) | Academic | No | - This quantitative cross-sectional study modified and used the COL core public health competencies to assess the training and educational needs of staff in a local health department. A total of 104 individuals from seven local health departments completed the assessment. - The COL-CCPH were used as "it is an approved framework for national public health accreditation for local health departments" (p81). - The authors noted that they did not receive any financial support for this work. |
| Cunningham | 2022 | Competency Status and Desire for Training in Core Public Health Domains: An Analysis by Job Level | Southwest, USA | This study examined the association between public health competency proficiency, desire for training, and current job level among nonmanagers (*n* = 790), middle managers (*n* = 332) and upper managers (*n* = 69) across 13 public health departments | Academic | No | - The COL-CCPH was adapted and used in this quantitative survey study. The study found gaps in competencies, particularly in those involving quantitative skills. The training most desired by respondents was leadership skills. - This research was funded through a cooperative agreement with HRSA. |

*List of abbreviations*:

ACGME-RRC Accreditation Council for Graduate Medical Education's Preventative Medicine Residency Review Committee

ANAPHI Australian Network of Academic Public Health Institutions

ASPH Association of Schools of Public Health

ASPHER Association of Schools of Public Health in the European Region

CAPHIA Council of Academic Public Health Institutions Australia

CCPH Core Competencies for Public Health

CDC Centers for Disease Control and Prevention

CEPH Council on Education for Public Health

COL Council on Linkages Between Academia and Public Health Practice

ECCMPHE European Core Competencies for MPH Education

HRSA Health Resources and Services Administration

LMIC Low- and middle-income country

MPH Master of Public Health

PHAC Public Health Agency of Canada

PHANZ Public Health Association of New Zealand

PHE Public Health England

PHF Public Health Foundation

PHSKF Public Health Skills and Knowledge Framework

UK United Kingdom

USA United States of America
