# Supplementary material for: The Governance of Core Competencies for Public Health: A Rapid Review of the Literature
Source: Public Health Rev. 2023 Sep 12;44:1606110. doi: 10.3389/phrs.2023.1606110 (PMC10520247; doi:10.3389/phrs.2023.1606110)
Supplement: Supplementary file 2 [file DataSheet1.docx]

**Supplementary File 1**: PubMed search strategy

**Date:** July 27, 2022

**Limits:** English; 2000-current

**Database:** PubMed

**Filters:** None

**Output Format:** RIS (first 500 from Best Match)

**Search Alerts:** None

**Concept #1: Competencies**

Professional Competence[majr] OR (competence[ti] OR competencies[ti] OR competency[ti] OR expertise[ti] OR skill[ti] OR skilled[ti] OR skills[ti] OR competence[ot] OR competencies[ot] OR competency[ot] OR expertise[ot] OR skill[ot] OR skilled[ot] OR skills[ot])

**Concept #2: Public Health**

American Public Health Association[majr] OR Education, Public Health Professional[majr] OR Environment and Public Health[majr] OR Nurses, Public Health[majr] OR Public Health[majr:noexp] OR Public Health Administration[majr] OR Public Health Dentistry[majr:noexp] OR Public Health Nursing[majr] OR Public Health Practice[majr:noexp] OR Public Health Systems Research[majr] OR Schools, Public Health[majr] OR Students, Public Health[majr] OR United States Public Health Service[majr:noexp] OR (public health*[ti] OR public health*[ot])

**Concept #3: Core**

(baseline[all] OR base-line[all] OR core[all] OR essential*[all] OR foundation*[all] OR framework*[all] OR frame-work*[all] OR generic*[all] OR practice*[all] OR federal*[all] OR headquarter*[all] OR head-quarter*[all] OR national*[all])

**Concept #4: Governance**

Evaluation Study[pt] OR "Organization and Administration"[majr] OR "Process Assessment, Health Care"[majr] OR Program Evaluation[majr] OR (administrat*[tiab] OR assess*[tiab] OR develop*[tiab] OR establish*[tiab] OR evaluat*[tiab] OR impact*[tiab] OR measur*[tiab] OR monitor*[tiab])

| **Search #** | **Query** | **Results** |
| --- | --- | --- |
| 7 | #1 AND #2 AND #3 AND #4, English, from 2000 - 2022/7/27 | 3,295 |
| 6 | #1 AND #2 AND #3 AND #4, English | 3,463 |
| 5 | #1 AND #2 AND #3 AND #4 | 3,629 |
| 4 | Evaluation Study[pt] OR "Organization and Administration"[majr] OR "Process Assessment, Health Care"[majr] OR Program Evaluation[majr] OR (administrat*[tiab] OR assess*[tiab] OR develop*[tiab] OR establish*[tiab] OR evaluat*[tiab] OR impact*[tiab] OR measur*[tiab] OR monitor*[tiab]) | 14,513,334 |
| 3 | (baseline[all] OR base-line[all] OR core[all] OR essential*[all] OR foundation*[all] OR framework*[all] OR frame-work*[all] OR generic*[all] OR practice*[all] OR federal*[all] OR headquarter*[all] OR head-quarter*[all] OR national*[all]) | 6,250,369 |
| 2 | American Public Health Association[majr] OR Education, Public Health Professional[majr] OR Environment and Public Health[majr] OR Nurses, Public Health[majr] OR Public Health[majr:noexp] OR Public Health Administration[majr] OR Public Health Dentistry[majr:noexp] OR Public Health Nursing[majr] OR Public Health Practice[majr:noexp] OR Public Health Systems Research[majr] OR Schools, Public Health[majr] OR Students, Public Health[majr] OR United States Public Health Service[majr:noexp] OR (public health*[ti] OR public health*[ot]) | 2,556,593 |
| 1 | Professional Competence[majr] OR (competence[ti] OR competencies[ti] OR competency[ti] OR expertise[ti] OR skill[ti] OR skilled[ti] OR skills[ti] OR competence[ot] OR competencies[ot] OR competency[ot] OR expertise[ot] OR skill[ot] OR skilled[ot] OR skills[ot]) | 126,392 |
